# Raltegravir 1200 mg once daily as maintenance therapy in virologically suppressed HIV-1 infected adults: QDISS open-label trial

**DOI:** 10.1186/s12981-022-00428-5

**Published:** 2022-01-15

**Authors:** Nolwenn Hall, Clotilde Allavena, Christine Katlama, Alexandra Jobert, Jean-Michel Molina, Eric Cua, Firouzé Bani-Sadr, Laurent Hocqueloux, Claudine Duvivier, Dominique Merrien, Hitoto Hikombo, Elisabeth André-Garnier, Aurélie Gaultier, François Raffi, the QDISS Study Group, Olivier Bollengier, Thomas Guimard, Sophie Leautez, Sophie Blanchi, Agathe Becker, Laurent Cotte, Tristan Ferry, Thomas Perpoint, Marie-Anne Trabaud, Laetitia Biron, Virginie Ferré, Laurent Flet, Véronique Reliquet, Audrey Rodalec, Christèle Volteau, Sophie Breaud, Pascal Puglièse, Eric Rosenthal, Barbara De Dieuleveult, Thierry Prazuck, Antoine Bachelard, Sylvie Legac, Yazdan Yazdanpanah, Jade Ghosn, Myriam Kalambay, Laurence Slama, Jean-Paul Viard, Jérémy Lourenco, Nadine Ktorza, Romain Palich, Luminita Schneider, Alexandre Aslan, Mariagrazia Tateo, Jeremy Zeggagh, Véronique Brodard, Maxime Hentzien, Isabelle Kmiec, Yohan N’Guyen, Faïza Ajana, Laurence Bocket, Thomas Huleux, Agnes Meybeck

**Affiliations:** 1 Public Health Center, CH Quimper, Quimper, France; 2 Department of Infectious Diseases, CHU Nantes, INSERM UIC 1413, CHU Nantes, Hôtel-Dieu University Hospital, 1 Place Alexis-Ricordeau, 44000 Nantes, France; 3 Department of Infectious Diseases, Assistance Publique-Hôpitaux de Paris, Pitié-Salpêtrière University Hospital, Paris, France; 4 Sponsor Department, CHU Nantes, Nantes, France; 5 Department of Infectious Diseases, Hôpital Saint-Louis, Assistance Publique-Hôpitaux de Paris, Université de Paris Diderot, Sorbonne Paris Cité, INSERM UMR 941, Paris, France; 6 Department of Infectious Diseases, Hôpital de l’Archet, Centre Hospitalier de Nice, Nice, France; 7 Department of Internal Medicine, Infectious Diseases, and Clinical Immunology, Reims Teaching Hospitals, University of Reims, Reims, France; 8 Department of Infectious Diseases, CHR d’Orléans, Orléans, France; 9 AP-HP-Necker Hospital, Infectious Diseases Department, Necker-Pasteur Infectiology Centre, Paris, France; 10 Department of Infectious Diseases, CHD Vendee, La Roche sur yon, France; 11 Infectious and Tropical Diseases Department, Le Mans General Hospital, Le Mans, France; 12 Virology Laboratory CHU Hôtel Dieu and INSERM CIC 1413 Nantes University, Nantes, France; 13 Department of Biostatistics, CHU de Nantes, Direction de la recherche, Nantes, France

**Keywords:** HIV-1 infection, Raltegravir, Once daily, Integrase inhibitor, Maintenance, Switch, Quality of life

## Abstract

**Background:**

Raltegravir (RAL) has favorable tolerability and safety profile, with few and manageable drug interactions. The use of RAL 1200 mg once daily (qd) for first-line therapy is well established. We assessed efficacy and safety of RAL 1200 mg qd, as part of triple combined antiretroviral therapy (cART), for maintenance strategy.

**Methods:**

The QDISS trial (NCT03195452) was a 48-week multicenter, single-arm, open-label study designed to evaluate the ability of 2 NRTIs + RAL 1200 mg qd to maintain virological suppression in HIV-1 infected subjects on a stable cART with 2 NRTIs and a third agent for at least 6 months. The primary endpoint was the proportion of participants with HIV-1 RNA < 50 copies/mL at week 24, by the FDA snapshot algorithm.

**Results:**

Of 100 participants 91% maintained viral suppression (95% CI: 83.6–95.8) at week 24 and 89% (81.2–94.4) at week 48. At week 24, there was one virological failure, without emergence of resistance-associated mutation and 10 participants had discontinued, 4 because of adverse events (AEs). Over 48 weeks, 7 AEs of grade 3–4 were reported, one possibly study-drug related (spontaneous abortion). BMI remained stable regardless of previous therapy or baseline BMI category. Over 48 weeks, total cholesterol (*p* = 0.023) and LDL-cholesterol (*p* = 0.009) decreased, lifestyle and ease subscale significantly improved (*p* = 0.04). The quality of life and Patients Reported Outcomes (PROs) also improved at W12 (*p* = 0.007).

**Conclusion:**

RAL 1200 mg qd as part of a maintenance triple therapy showed a high efficacy in virologically suppressed HIV-1 infected subjects, with good safety profile and improved lipid profile and patient reported outcomes.

*Trial registration:* Clinical trials.gov NCT03195452 and EudraCT 2016-003702-13.

## Background

As viral suppression is achieved in most people living with HIV (PLWHIV) with currently recommended regimens, HIV treatment goals has shifted towards a durable viral suppression and an optimal long-term tolerability of cART with regimens that are life-style compatible [1]. This is even more relevant in the context of Western countries, where approximately half of PLWHIV are older than 50 years, with a growing tribute of age-related and non-HIV disease-related comorbidities, associated medications, and possible effects of long-term chronic exposure to cART [2]. Integrase strand transfer inhibitors (INSTIs), given their high antiviral potency and their favorable safety profile are now recommended, as the cornerstone of cART, in all HIV infected individuals [3]. Although bictegravir and dolutegravir are preferred among the INSTI class, there are not free of some concerns, such as weight gain, emergence of metabolic abnormalities and neuropsychiatric adverse events. Raltegravir (RAL), the first INSTI, has high virological potency together with low drug-drug interaction, good safety profile, such as lipid, inflammation and bone parameters [4], but the twice-per-day (bid) dosing was a real constraint in the era of treatment simplification. In 2017, a new formulation of RAL 1200 mg (2 × 600 mg tablets) qd showed non inferior efficacy and similar safety to RAL 400 mg bid for previously untreated HIV-1 patients, in combination with other antiretroviral agents [5]. As no study has yet assessed the efficacy of a switch to RAL 1200 mg qd in patients virologically suppressed, we aimed, in this open-label, single-arm study, to assess the capacity of RAL 1200 mg qd combined with 2 nucleoside reverse transcriptase inhibitors (NRTIs) to maintain virological success in patients virologically suppressed.

## Methods

### Study design and participants

QDISS study was a 48 weeks open-label, single-arm, prospective study, conducted in 17 clinical sites in France, designed to evaluate the ability of 2 NRTIs in association with RAL qd to maintain virological suppression in HIV-1 infected adults receiving a stable therapy with 2 NRTIs (tenofovir disoproxil fumarate (TDF)/ /lamivudine (3TC), tenofovir alafenamide (TAF)/emtricitabine (FTC) or abacavir (ABC)/3TC) and a third agent (including RAL 400 mg bid but less than 30% of the total cohort), for at least 6 months. Adults aged 18 years or older were eligible if they had plasma HIV-1 RNA < 50 copies/mL for at least 6 months with no more than one blip (between 50 and 200 copies/mL) and no prior virological failure on INSTI or non-nucleoside reverse transcriptase inhibitor- based cART or on NRTI(s) only-therapy. Additional inclusion criteria included absence of resistance to any antiretroviral drug except E138A/G/K/Q/R/S or V179D in blood HIV-RNA or DNA, aspartate and alanine aminotransferase concentration below five times the upper limit of normal, haemoglobin > 8 g/dl, platelet count > 50 000/mm3, estimated glomerular filtration rate by MDRD equation > 50 mL/min. Women of childbearing potential had to have a negative pregnancy test at screening and to accept the use of contraceptive methods during the study. All participants had an indication to change their current triple cART for intolerance or prevention of toxicity, or presence of comorbidity justifying change of the 3^rd^ agent, or management of drug-drug interaction, or for patient’s request to simplify or to improve regimen convenience. Patients with chronic hepatitis B infection were allowed in the study if their treatment included TDF or TAF.

### Ethical considerations

The study was conducted according to Good Clinical Practice. The protocol was approved by the Ethics Committee of Est III (17.04.06), and authorized by the French National Agency for the Safety of Medicines (ANSM 170133A-41). All participants gave written informed consent. The study was registered with ClinicalTrial.gov (NCT03195452).

### Study procedures

After enrolment, participants switched to RAL 1200 mg qd, while maintaining the same background 2 NRTIs. Participants were evaluated at baseline, weeks 4, 12, 24, 36, and 48. At all study visits, assessments were done for symptom-directed physical examinations, weight, adverse events and concomitant medications, and symptom-directed physical examinations were done. Laboratory tests included haematological analysis, serum chemistry and measures of plasma HIV-1 RNA at each visit, fasting glucose and lipid parameters and CD4 and CD8 cell counts at baseline, weeks 36 and 48. Protocol-defined virological failure (PDVF) was occurrence of 2 consecutive HIV-1 RNA ≥ 50 copies/mL, the second specimen being taken within 1 to 4 weeks. Genotypic resistance testing was performed in case of PDVF on the confirmatory sample and interpreted following the ANRS algorithm for drug resistance-associated mutations, version 27 (http://www.hivfrenchresistance.org). Adherence was assessed by visual scale, at day 0, weeks 24 and 48. Treatment satisfaction was assessed using two versions of the revised HIV Treatment Satisfaction Questionnaire (HIVTSQ): the HIVTSQs (status version) at baseline, week 24 and 48 and the HIVTSQc (change version) at week 24 [6, 7]. Two subscale measured general satisfaction/clinical and lifestyle/ease satisfaction. The HIVTSQs measured change in within-participant treatment satisfaction over time, a higher score indicating greater improvement in treatment satisfaction (total treatment score ranging from 0 to 66). The HIVTSQc compared participants’ views of their current RAL 1200 mg qd-including therapy with their prior treatment (score range from − 33 to 33). Health-related quality of life was assessed by the PROQOL-HIV self-administered questionnaire [8], at day 0, weeks 12 and 48.

### Study endpoints

The primary endpoint was the proportion of participants with HIV-1 RNA < 50 copies/mL at week 24, by the US FDA snapshot algorithm. This endpoint was set at W24 in order to discuss the possibility to halt the study if the lower 95% CI of the success rate was < 80%. Secondary endpoints were safety and tolerability, virological suppression at week 48, emergence of resistance mutations in case of PDVF, adherence, patient satisfaction, quality of life and patient reported outcomes (PROs).

### Statistical analysis

A sample size of 94 patients was required to give a 85% probability of rejecting a baseline response rate of 80% with an exact 5% one-sided significance test when the true response rate is 90%. To take into account patients with consent withdrawal or lost to follow-up (5%), 100 patients had to be enrolled. All patients who received at least one dose of study medication were included in the analysis. No data imputation was performed for secondary endpoint analysis. Baseline variables were described, for all patients, by numbers and percentages for categorical variables and means and standard deviation (sd) for quantitative variables. The proportion of patients with plasma HIV-1 RNA < 50 copies/mL, by the US FDA snapshot algorithm, at week 24 was estimated with 95% exact confidence interval. Changes in biological parameters or questionnaire scores were analysed using paired Student's tests. All analy﻿ses were conducted with software R v3.6.0 (R Foundation for Statistical Computing, Vienna, Austria).

## Results

**Fig. 1:**
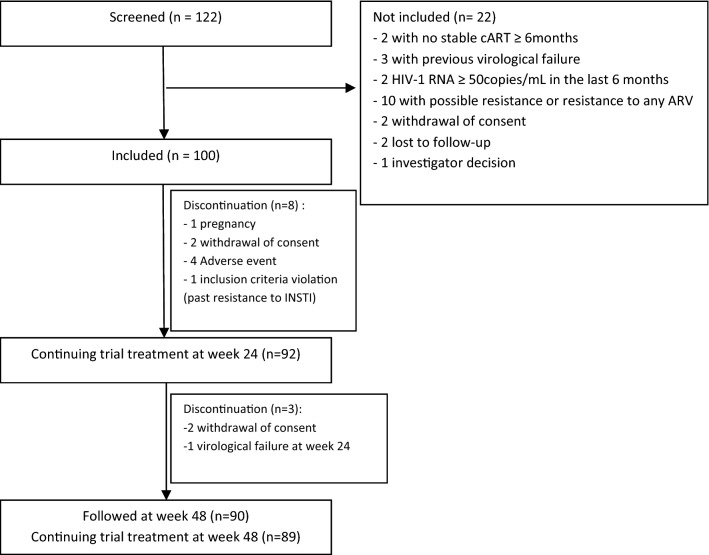
Study flow chart

From November 2017 to April 2019, 100 subjects were enrolled out of 122 screened individuals, in 17 centers (Fig. 1). Participants’ baseline characteristics are shown in Table 1. At screening, 18% of participants were receiving a single tablet regimen. Reason for switching their current cART were intolerance or prevention of toxicity in 34% of participants, presence of comorbid condition justifying change of the 3rd agent in 13%, drug-drug interactions in 4% and patient’s request in 52%. The proportion of patients who maintained HIV-1 RNA < 50 copies/mL in the ITT analysis was 91% (95% CI: 83.6–95.8) at week 24, and 89% (81.2–94.4) at week 48. Overall, only one subject experienced PDVF, at week 24, with plasma HIV-1 RNA of 69,000 and 290 copies/mL on first and confirmatory samples, respectively. On confirmatory sample, there was no emergence of resistance-associated mutation to RAL or NRTIs on genotype testing. Over the 48 weeks of the study, 10 treatment discontinuations occurred for reasons other than PDVF (consent withdrawal, n = 4, major violation of entry criteria, n = 1, pregnancy, n = 1, adverse event, n = 4). Thus, the success rate of the strategy was 94.7% (95% CI = 88.0–98.3) at week 48.

**Table 1:** Baseline characteristics of the patients

Characteristic	n = 100
Male, n (%)	77 (77)
Age (years), median (IQR)	47 (40–58)
Origin, n (%)	
Caucasian	61 (61%)
Sub-saharian africa	32 (32%)
Others	7 (7%)
Time since HIV diagnosis (years), median (IQR)	10.3 (5–17)
CDC classification, n (%)	
A	62 (62%)
B	14 (14%)
C	24 (24%)
CD4 nadir cell count (cells/mm^3^), median (IQR), n = 99	233 (122–372)
CD4 cell count at screening (cells/mm^3^), median (IQR)	642 (473–875)
Antiretroviral therapy duration (years), median (IQR)	8.5 (4.7–14.5)
Duration of last ARV therapy (years), median (IQR)	3.3 (1.6–5.6)
ARV therapy at screening, n (%)	
2 NRTIs + Protease inhibitor	40 (40%)
2 NRTIs + 1 NNRTI	13 (13%)
2 NRTIs + 1 INSTI	47 (47%)
2NRTIs + RAL BID	32 (32%)
Active hepatitis co-infection, n (%)	
Hepatitis B virus (Positive HBs Ag)	5 (5%)
Hepatitis C virus (Positive RNA)	0 (0%)
BMI (kg/m^2^), mean (± sd)	26.6 (5.6)
Creatinine (µmol/l), mean (± sd), n = 99	82 (± 17.12)
eGFR (MDRD) (mL/min), mean (± sd), n = 99	95.70 (± 23.67)
Total cholesterol (mmol/L), mean (± sd), n = 98	4.76 (± 0.95)
LDL cholesterol (mmol/L), mean (± sd), n = 95	2.86 (± 0.73)
HDL cholesterol (mmol/L), mean (± sd), n = 96	1.32 (± 0.54)
Triglycerides (g/l) mean (± sd), n = 98	1.43 (± 0.88)
Glycaemia (mmol/L), mean (± sd), n = 85	5.33 (± 1.28)

During the total 48 weeks follow-up, a total of 7 adverse events of grade 3–4 were reported, one possibly related to study drug (spontaneous miscarriage at week 8); 4 of these 7 events were classified as serious (acute HCV infection (1), traumatic bone fracture (1), hematemesis (1), miscarriage (1)). Most frequent AEs, occurring in > 10% of participants, were gastrointestinal, musculoskeletal, neuropsychiatric disorders, infections and infestations, and respiratory disorders. Reasons for discontinuation because of treatment-related adverse events were gastro-intestinal side effects (n = 2, at week 1 and week 4), gastrointestinal disorder and headache (n = 1, at week 6), insomnia, nightmare and arthralgias (n = 1, at week 4). BMI remained stable (mean change at W48: 0.15 (± 1.23) *p* = 0.28) regardless of previous therapy (RAL or ARV therapy without RAL) or baseline BMI category. Mean total cholesterol and LDL-cholesterol significantly decreased at W48 (− 0.21 mmol/L (± 0.84), *p* = 0.023; and − 0.19 mmol/l (± 0.66) *p* = 0.009, respectively). There were no significant changes in triglycerides, HDL-cholesterol and glycaemia at week 48 (mean change: − 0.08 (± 0.67), *p* = 0.3; 0.01 (± 0.26), *p* = 0.61, and 0.22 (± 1.03), *p* = 0.081, respectively). Renal parameters were unchanged. Median increase in CD4 cell count at week 48 was 50 cells/mm [3] (IQR: − 33 to 142; *p* < 0.001).

Adherence to cART was high (97% intake) and stable throughout the 48 weeks follow-up. At baseline, patient global satisfaction (evaluated by the HIVTSQs) was high and remained unchanged until week 48 (mean change = 0.81 ± 9.06) *p* = 0.42). The lifestyle and ease subscale significantly improved between baseline and week 48 (mean change = 0.88 ± 3.86); *p* = 0.04. Participants mostly preferred the new regimen, with a total satisfaction change score (HIVTSQc) of 21.3 (± 11.79) at week 24. The quality of life and PROs, improved from baseline to week 12 with total score of 47/100 (± 15.10) and 50/100 (± 14.46) respectively (*p* = 0.007). There was also improvement in the body change dimension (*p* = 0.034), and the treatment impact dimension (*p* = 0.001). No significant difference was observed for other PROs, and between baseline and week 48, for any PRO.

## Discussion

Our study showed the high efficacy and safety of switching to a triple therapy based on 2 NRTIs plus RAL 1200 mg qd in virologically suppressed patients. At both weeks 24 and 48, maintenance of HIV-1 RNA < 50 copies/mL by FDA snapshot analysis was around 90% with the lower margin of 95% CI above the predefined 80% value. RAL 1200 mg qd demonstrated non-inferiority to RAL 400 mg bid, each combined with emtricitabine and tenofovir disoproxil fumarate in previously untreated HIV-1 patients [5]. Despite high virological efficacy, irrespective of baseline viral load, excellent tolerability and safety profile, and low potential for drug interactions, the small risk of resistance emergence (0.2% to 1.4% of patients in RAL 400 mg bid studies [5, 9, 10], and in 0.9% of patients with RAL 1200 mg qd [11], at week 96) has led to prefer more recent INSTIs, namely bictegravir and dolutegravir for first-line cART [1, 2]. In virologically suppressed patients, bictegravir-based and dolutegravir-based combinations have also demonstrated very high efficacy and good safety [12, 13]. The SWITCHMRK and SPIRAL studies enrolled patients having sustained virological suppression on ritonavir-boosted protease inhibitor-based therapy with or without prior virological failure and demonstrated that in those with no history of virological failure, switching to RAL 400 mg bid, while continuing background therapy, was associated with > 90% rate of maintenance of virological suppression [14, 15]. No INSTI resistance was evidenced after 48 weeks of switch to RAL 400 mg bid in the SPIRAL study [15]. Our study is the first to show the high efficacy of switching to RAL 1200 mg qd plus 2 NRTIs in virologically suppressed patients with no history of prior virological failure to NRTI-based regimens. Overall, strategy success of the 2 NRTIs plus RAL 1200 mg qd switch was 95% at week 48, with only 1% of virological failure, concomitant with a poor transient non-adherence without emergence of resistance, and 4% of discontinuation for low grade adverse events. Of note, there was only 1 case (1%) of discontinuation for neuropsychiatric disorder. Discontinuation of RAL bid because of neuropsychiatric adverse events has been reported to be < 1% [16]. This is lower than the rate of discontinuation for neuropsychiatric adverse events with dolutegravir plus 2 NRTIs, that has been reported between 1.7 and 6% at 1 year [16, 17]. In addition, there was no significant weight or BMI changes after RAL 1200 mg qd switch and fasting lipid total cholesterol and LDL-cholesterol improved, confirming the neutral metabolic profile of RAL compared to protease inhibitors or TAF-containing regimens [18, 19]. Issues on weight gain increase have been recently raised following bictegravir or dolutegravir initiation, especially in previously untreated persons but also in the setting of switch for maintenance, while RAL has minimal effect [20, 21]. No BMI change was seen in the subgroup of patients switched form a non-RAL regimen, adding further evidence of differentiation of RAL within the INSTI class. This should be interpreted cautiously as, in our study, participants were mainly Caucasian male with a normal baseline BMI. Improvement in PROs and of quality of life are particularly noteworthy, in the context of chronic asymptomatic disease, in relation to the good tolerability of RAL 1200 mg qd, even though this regimen implies 3 pills once a day. Participants also mostly preferred their new regimen.

Our study have some limitations, including the absence of a control group, which in terms of impact on metabolic parameters (lipids, glycemia, BMI) may limit the interpretation of the observed changes over time. Although absence of a comparator group precludes any formal conclusion, efficacy rate of 89% is at the same level as for other INSTIs as maintenance switch strategy. Second, the magnitude of patients’ satisfaction might have been biased by the open label design of the study and by the request for a change of cART by patients themselves in 52% of cases. Also, because of the diversity of prior cART regimen, we could not assess changes in PROs according to previous regimen.

In conclusion, results of our study position RAL 1200 mg qd as an alternative option to bictegravir or dolutegravir as INSTI plus 2 NRTIs switch in virologically suppressed patients.

## Data Availability

The data that support the findings of this study are available from CHU Nantes but restrictions apply to the availability of these data, which were used under license for the current study, and so are not publicly available. Data are however available from the authors upon reasonable request and with permission of CHU Nantes.
